# The Implementation and Evaluation of Health Promotion Services and Programs to Improve Cultural Competency: A Systematic Scoping Review

**DOI:** 10.3389/fpubh.2017.00024

**Published:** 2017-02-27

**Authors:** Crystal Sky Jongen, Janya McCalman, Roxanne Gwendalyn Bainbridge

**Affiliations:** ^1^School of Human Health and Social Sciences, Central Queensland University, Cairns, QLD, Australia

**Keywords:** cultural competency, indigenous, ethnic minorities, health disparities, health promotion, program implementation

## Abstract

**Background:**

Cultural competency is a multifaceted intervention approach, which needs to be implemented at various levels of health-care systems to improve quality of care for culturally and ethnically diverse populations. One level of health care where cultural competency is required is in the provision of health promotion services and programs targeted to diverse patient groups who experience health-care and health inequalities. To inform the implementation and evaluation of health promotion programs and services to improve cultural competency, research must assess both intervention strategies and intervention outcomes.

**Methods:**

This scoping review was completed as part of a larger systematic literature search conducted on evaluations of cultural competence interventions in health care in Canada, the United States, Australia, and New Zealand. Seventeen peer-reviewed databases, 13 websites and clearinghouses, and 11 literature reviews were searched. Overall, 64 studies on cultural competency interventions were found, with 22 being health promotion programs and services. A process of thematic analysis was utilized to identify key intervention strategies and outcomes reported in the literature.

**Results:**

The review identified three overarching strategies utilized in health promotion services and programs to improve cultural competency: community-focused strategies, culturally focused strategies, and language-focused strategies. Studies took different approaches to delivering culturally competent health interventions, with the majority incorporating multiple strategies from each overarching category. There were various intermediate health-care and health outcomes reported across the included studies. Most commonly reported were positive reports of patient satisfaction, patient/participant service access, and program/study retention rates. The health outcome results indicate positive potential of health promotion services and programs to improve cultural competency to impact cardiovascular disease and mental health outcomes. However, due to measurement and study quality issues, it is difficult to determine the extent of the impacts.

**Discussion:**

Examined together, these intervention strategies and outcomes provide a framework that can be used by service providers and researchers in the implementation and evaluation of health promotion services and programs to improve cultural competency. While there is evidence indicating the effectiveness of such health promotion interventions in improving intermediate and health outcomes, further attention is needed to issues of measurement and study quality.

## Introduction

The 1986 Ottawa Charter for Health Promotion set out a broad vision and inclusive framework to guide the global movement for advancing health equity (1). The Charter recognized the importance of advocating, enabling, and mediating for improved health across sectors and groups in society. Today, in societies of significant and growing sociocultural diversity with accompanied health inequalities between population groups, the call for sustained, broad-reaching, and coordinated efforts to work toward health equity are needed as much as they were 30 years ago. The Charter emphasized that “health promotion strategies and programmes should be adapted to the local needs and possibilities … and take into account differing social, cultural and economic systems” (1). Yet despite this call for local adaptation, many health promotion strategies and programs have failed to design programs for populations with complex and diverse needs that reflect social and cultural realities and are meaningful to people’s health practices and beliefs (2). The challenge of developing and implementing programs which meet culturally diverse community needs while simultaneously drawing out and enhancing individual and community assets is of utmost importance to the development of health promotion globally (3). One way this has been addressed in health care is through the provision of direct health promotion services and programs which aim to be culturally competent for the targeted communities and populations.

Cultural competency emerged as a strategy to address cultural differences between health practitioners and patients who had health beliefs and practices which differed to those common in western bio-medicine. In this context, cultural competency was concerned with interpersonal dynamics in health-care encounters and how cultural dissonance can impact patient outcomes (4). However, over time, cultural competency evolved into the recognition that to enable truly culturally appropriate health care for diverse population groups, it is necessary that all levels of health services and systems be embedded in culturally competent frameworks (4). This requirement for cultural competency to be integrated across all levels of health-care systems is reflected in the definition of cultural competency provided by Cross et al. (5). Cross et al. define cultural competency “as a set of congruent behaviors, attitudes and policies that come together in a system, agency or among professionals that enable that system, agency or professions to work effectively in cross-cultural situations” (5). Today, cultural competency includes diverse intervention strategies targeting not only health practitioners but also whole organizations and health systems (6).

**Figure 1:**
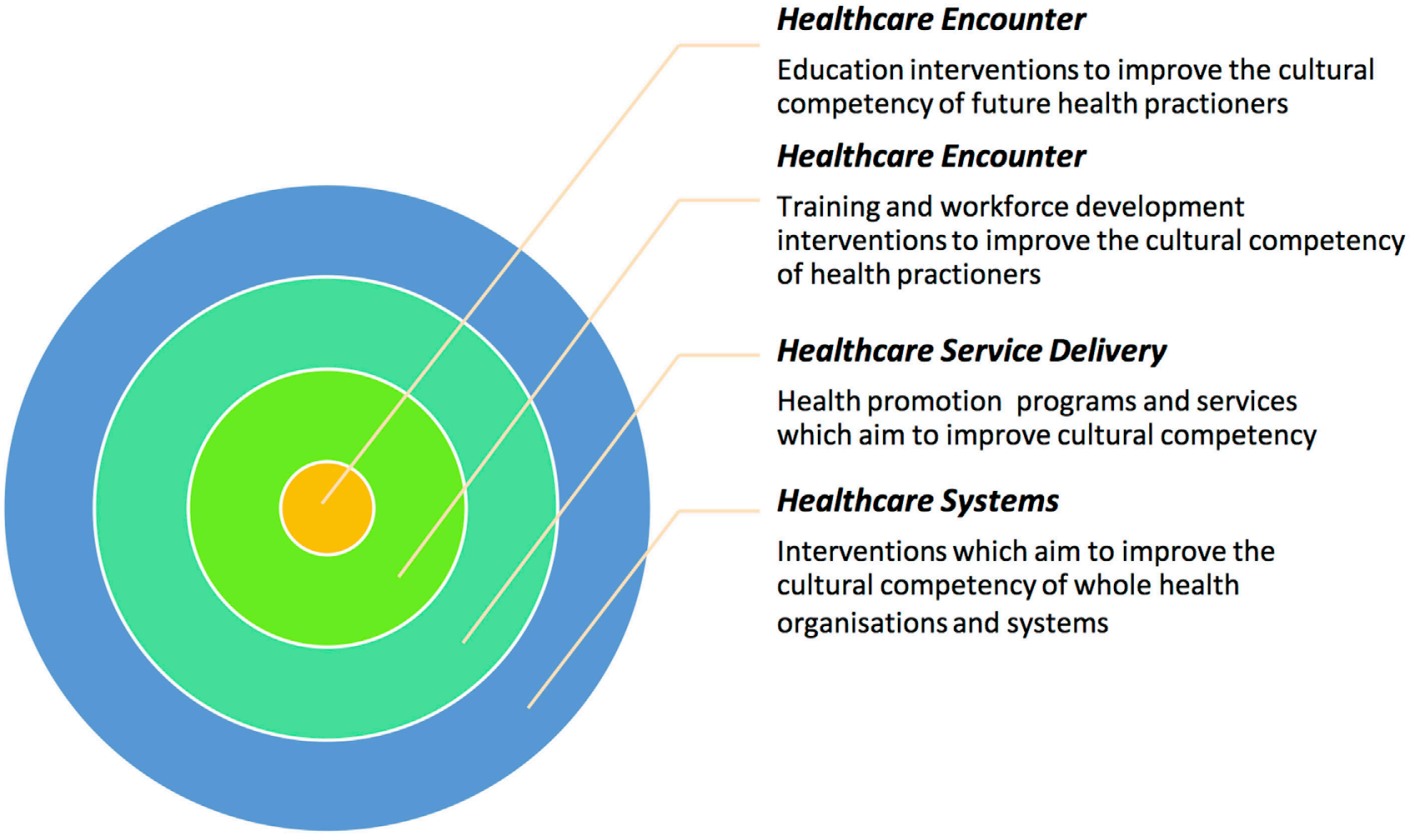
**Systems analysis of cultural competency in healthcare**.

Despite these developments in the conceptualization and scope of cultural competency, there is no common framework of cultural competency for use across different health contexts either within or between countries (6). The cultural competency literature reveals great variation in interventions that aim to improve cultural competence in health care. One factor contributing to this variation is the level of health-care systems toward which interventions are aimed. A recent review of cultural competency intervention studies has identified that cultural competency interventions are generally implemented on one of four levels across health-care services and systems (7). The first two levels are concerned with the health-care encounter and address the cultural competency of individual health practitioners and professionals. One set of interventions target health students while studying and training, and the other targets health professionals working in the field. In both of these intervention sets, the focus is on teaching the requisite knowledge, attitudes, skills, and behaviors needed to ensure safe and effective health care for diverse patient groups. The next level is concerned with health-care service delivery. In this level, the appropriateness or cultural competence of health programs and services themselves is the focal point. Finally, encompassing all other levels are those interventions concerned with health-care systems. These interventions aim to improve the cultural competence of entire health organizations and systems (see Figure 1 for an illustration of this multileveled approach to understanding cultural competency).

The intervention strategies employed in each level are necessarily distinct, as too are the measurement tools applied to evaluate intervention impacts (8). Subsequently, confusion can be caused when different approaches toward addressing cultural competency on different health-care levels are examined together. To accurately conceptualize, implement, and evaluate cultural competency, a multiple system-level framework is needed that addresses the unique characteristics of intervention and evaluation approaches appropriate to the different levels on which it operates. This review is concerned with cultural competency interventions implemented on the health service or program level. The goal of improving the cultural competency of health promotion services and programs is premised on the recognition that no single approach to providing health care fits all people and populations (9). In this level, cultural competency interventions focus on improving health and well-being through the integration of cultural understanding and responsiveness into health services delivery (10). In this paper, these health promotion interventions will be referred to as cultural competency services and programs.

Reviews of the literature on cultural competency services and programs primarily aim to build the evidence base of what approaches work best for whom, by focusing on the outcomes of interventions. Previous research has shown promising evidence of the success of service-level cultural competency interventions on certain intermediate health-care and health outcomes (6, 11). Truong et al. (6) completed a systematic review of literature reviews on interventions to improve cultural competency in health care. Patient/client outcomes reported across reviews examined included improvements in patient knowledge, lifestyle and dietary behaviors, and certain clinical outcomes, such as glycemic control for diabetes patients (6). However, study quality and measurement issues limited confidence in the evidence that cultural competency improves health outcomes (6).

Although less attention has been given to intervention strategies, previous literature reviews have outlined various approaches taken to address cultural competency in health services and programs. In a 2000 literature review by Brach and Fraser, a range of intervention strategies commonly applied to improve the cultural competency of services and programs were identified. These strategies included coordinating with traditional healers, the inclusion of family and community members, the involvement of community health workers, and the use of interpreter services (9). Later, Goode et al. found that while there was no consistent model used for developing cultural competency services and programs, strategies utilized included community input; health information delivered by community members; adapting intervention delivery modes; ensuring language access through bilingual or bicultural staff and materials in preferred languages; and alignment of interventions with cultural beliefs, values, and practices (11). While these strategies are discussed, there have been no attempts in the literature to date to develop a framework of intervention strategies, which can be used to integrate cultural competence in health service/program planning and implementation. To this end, this literature review has a dual focus. It aims to (1) inform the planning and implementation of cultural competency services and programs by reviewing interventions strategies; and (2) inform further evaluations of cultural competency services and programs to help improve the quality of the evidence base by reviewing outcomes, measurement tools, and reporting on study quality.

## Methods

This scoping review is extracted from a broader rapid systematic review, commissioned by the SAX Institute and Closing the Gap Clearinghouse in Australia, to identify publications on cultural competency interventions in health care for indigenous peoples and other minority ethnic/cultural groups in Australia, New Zealand, Canada, and the United States. The broader review aimed to determine the intervention strategies and indicators which have been applied to increase cultural competency in health care, along with the outcomes of these interventions.

## Inclusion/Exclusion Criteria

Studies in this review included peer-reviewed and gray literature published in English from January 1, 2006 to December 31, 2015 inclusive. The start date of the review was taken from 2006 following the United States comprehensive review of cultural and linguistic competence in health care by Goode et al. (11). Publications were included if the following criteria were met:

1. The study was from Australia, Canada, New Zealand, or the United States;
2. The study was focused on cultural competence as it pertains to indigenous or other racial or ethnic groups; and
3. The study evaluated an intervention designed to improve cultural competence in health care.

## Search Strategy

**Figure 2:**
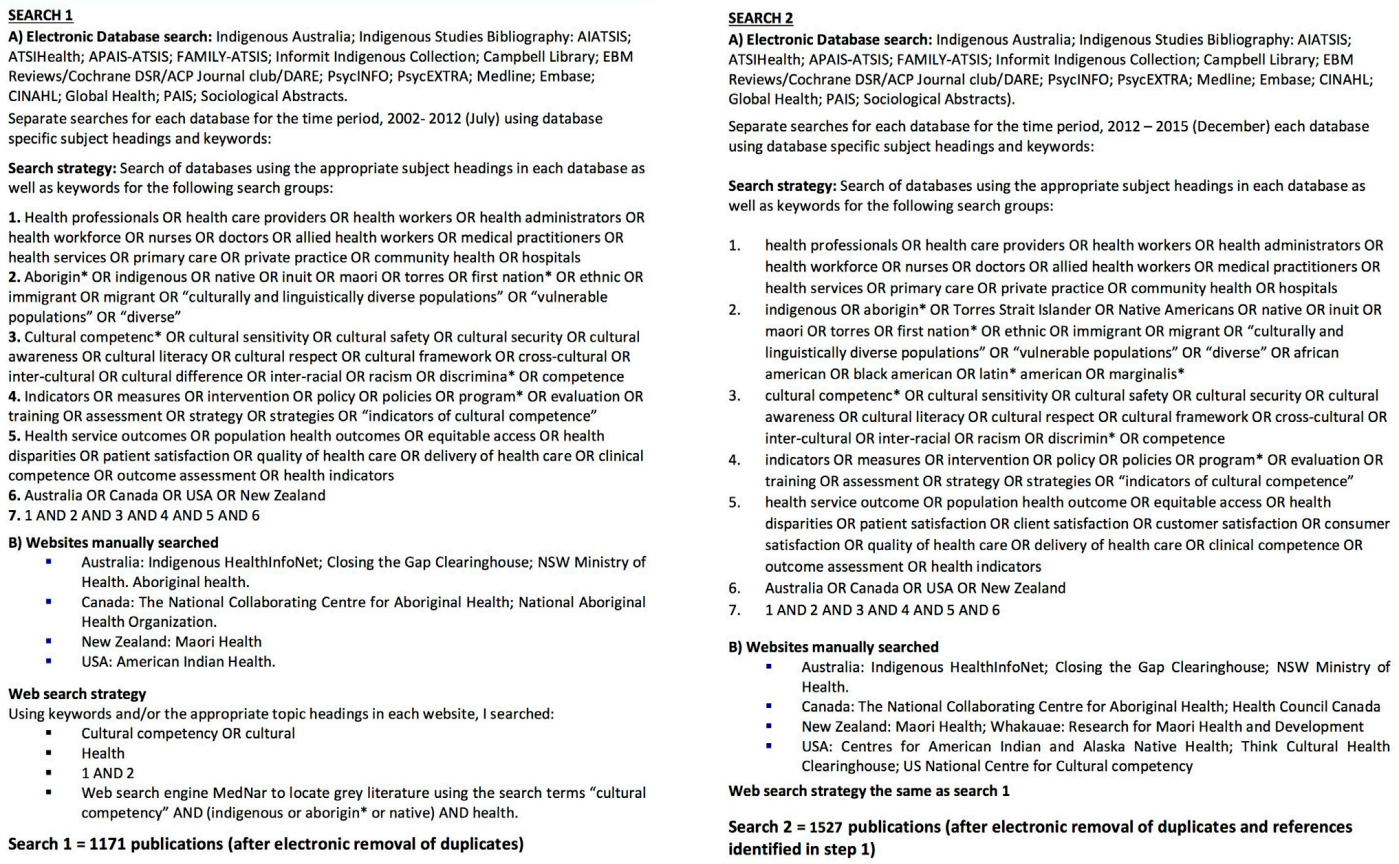
**Search strategies 1 and 2**.

The search strategy employed for the review comprised six steps covering an initial search in 2012, for the period 2002–July 2012, and a search update in 2016 for the period 2012–2015. See Figure 2 for summaries of search 1 and search 2.

**Figure 3:**
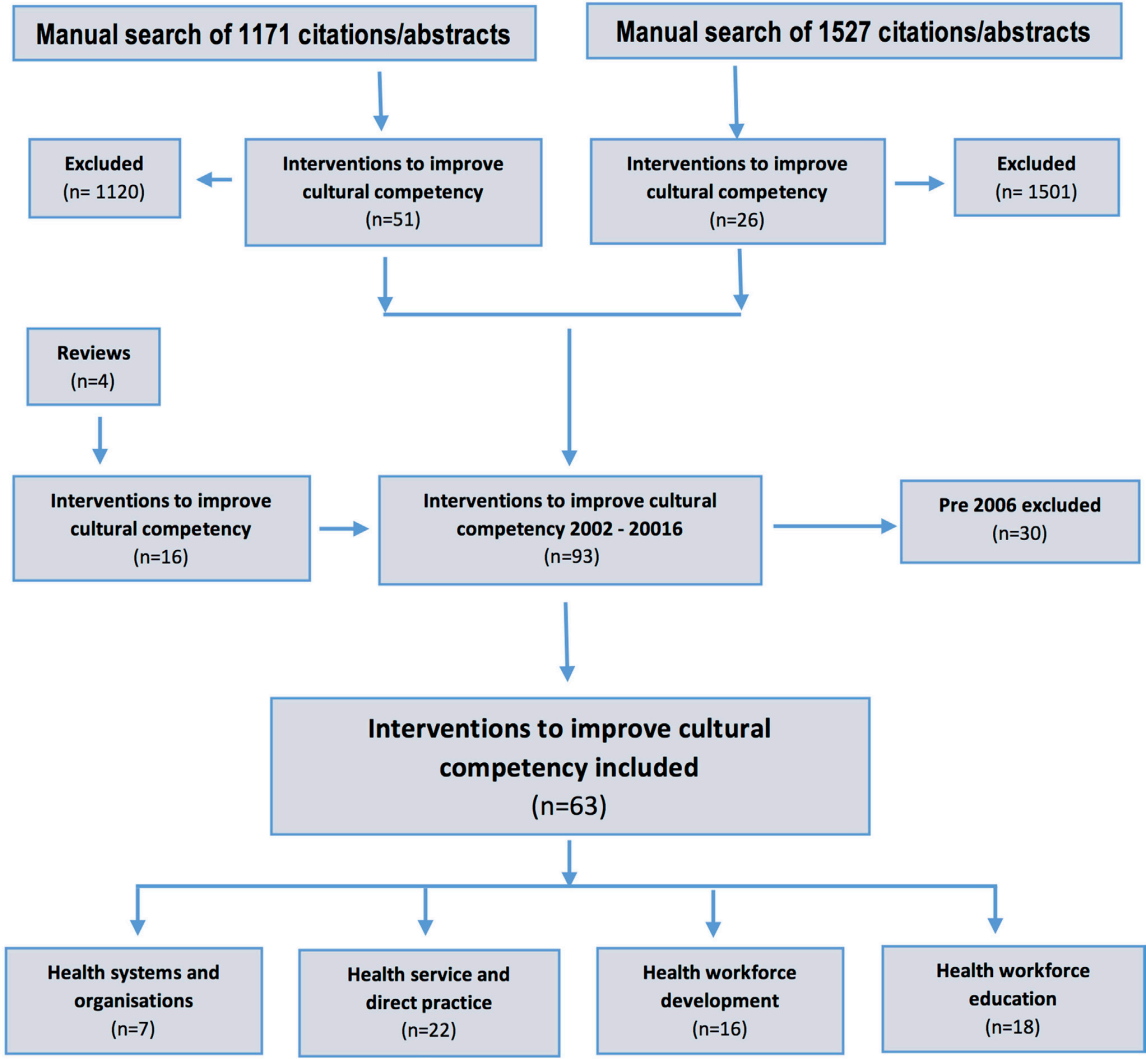
**PRISMA search strategy flow chart**.

*Step 1*: an expert librarian (Mary Kumjav) searched 17 relevant electronic databases identifying 1,135 references excluding duplicates.
*Step 2*: relevant gray literature in clearinghouses and websites of relevant organizations in each of the four countries were searched for additional literature, locating 30 more publications.
*Step 3*: the reference lists of seven reviews were examined manually locating an additional six studies for inclusion.
*Step 4*: the 1,171 references identified were imported into Endnote and their abstracts manually examined, with 51 intervention studies meeting the inclusion criteria.
*Step 5*: steps 1–4 were repeated again in June 2016 in a search update. The search terms used were modified slightly to try to capture further relevant literature, and some websites previously searched were no longer operational so other websites and clearing houses were identified (see Figure 1). The updated search identified 1,511 references from the electronic database search and an additional 16 from the gray literature. The 1,527 references identified were imported into Endnote and their abstracts examined manually. There were 26 intervention studies that met the inclusion criteria. The reference lists of an additional 4 literature reviews revealed 16 more studies to be included.
*Step 6*: given the availability of the Goode et al.’s (11) review, it was decided following the updated search to include only studies published between the years 2006 and 2016. A total of thirty studies were excluded at this stage, leaving 63 total studies for inclusion. See Figure 3 for PRISMA search strategy flow chart (12).

## Identification, Screening, and Inclusion of Publications

The search results of both searches were imported into the bibliographic citation management software, Endnote X7 with duplicates removed. Titles and abstracts of the remaining publication titles and abstracts in the first search were screened by one author (Roxanne Gwendalyn Bainbridge). A second author (Crystal Sky Jongen) retrieved and screened titles and abstracts of the remaining publications from the second search; those which did not meet inclusion criteria were excluded. The full texts of the remaining publications were retrieved and screened by blinded reviewers (Roxanne Gwendalyn Bainbridge, Janya McCalman, Anton Clifford, and Komla Tsey). Inconsistencies in reviewer assessments were resolved by consensus.

## Data Extraction and Analysis

Data were extracted from the full texts of studies for publication authorship, year and type, country, population and sample size, intervention setting, intervention type and strategies, study design, outcome measures, and outcomes reported (the full data extraction table for the included studies can be found in the supplementary material). The quality of included quantitative studies was assessed using the Effective Public Health Practice Project quality assessment tool (13). Qualitative studies were assessed using the Critical Appraisal Skills Programme quality assessment tool (14).

Of the 63 studies included in the larger review, 22 studies evaluated cultural competency interventions operating at the direct practice or service level of health promotion and treatment targeting specific population groups. Studies were classified as addressing cultural competency at the service level if they met one of the following criteria: assessed satisfaction levels (patient and health staff); assessed acceptability; assessed appropriateness; assessed feasibility; included a comparison or control; directly assessed cultural competency or related terms, or; reported on the development of cultural competency interventions. Thematic analysis methods (15) were used to identify key themes across evaluations. A mind map was constructed to sort the intervention strategies utilized and their associated outcomes. Overarching themes were then reviewed, refined, and named (15).

## Results

Twenty-two studies of cultural competency services and programs were included. The evaluation studies by country, target group, and health issue are shown in Table 1. The results from the included studies are assessed by both intervention strategies utilized and intervention outcomes reported (see Table 2 for an overview).

**Table 1:** **Studies by country, target population, and health issue**.

Country	Target population	Health issue addressed					Total
		Mental health	Diabetes	Cancer	Cardiovascular disease	Others	
United States		4	2	4	2	1	13
	Native American			2	1	1	4
	African-American	1		1	1		3
	Latino American	1	2				3
	Native Hawaiian			1			1
	Haitian American	1					1
	Chinese American	1					1
Australia							6
	Australian indigenous	1	1			4	6
Canada							2
	First Nations Canadian		1				1
	South East Asian Canadian				1		1
New Zealand							1
	Asian New Zealander					1	1
Total		5	4	4	3	6	22

**Table 2:** **Intervention strategies and outcomes**.

		Community strategies				Culture strategies				Language strategies			Intermediate outcomes						Health outcomes
						
Reference	Increases cultural competency	Community participation	Community partnerships	Community spaces	Community networks	Values/beliefs/practices	Activities	Religion/spirituality	Interactive/visual resources	Full language adaptation	Partial language adaptation	Written/audiovisual	Patient perceived satisfaction	Health worker acceptability	Service utilization/access	Retention/adherence/treatment	Improved health knowledge/	Improved behavioral outcomes	Improved health status outcomes
Arora et al. (17)	✓	✓	~	✓	x	✓	✓	✓	x	✓	x	x	✓	~	✓	x	~	x	x
Browne et al. (35)	✓	x	x	x	x	x	x	x	✓	x	x	x	x	✓	x	x	~	x	x
Chavez-Korell et al. (32)	✓	✓	✓	✓	✓	✓	✓	✓	x	✓	x	✓	✓	x	x	✓	~	✓	✓
Davies et al. (16)	✓	✓	✓	x	x	x	x	x	✓	x	x	✓	✓	x	x	x	✓	x	x
Dingwall et al. (20)	✓	✓	~	x	x	✓	x	~	✓	x	x	x	x	✓	x	x	x	x	x
D’Silva et al. (34)	✓	✓	~	✓	x	✓	✓	✓	x	x	✓	x	x	x	~	~	x	✓	x
Guadagnolo et al. (27)	✓	✓	~	x	x	✓	x	x	x	x	x	✓	✓	x	x	x	x	x	x
Houston et al. (37)	~	✓	x	x	x	x	x	x	x	x	x	x	x	x	x	x	x	x	✓
Jandorf et al. (22)	✓	✓	x	x	x	✓	x	x	x	x	✓	x	✓	x	~	✓	x	x	x
Jones et al. (24)	✓	✓	✓	✓	✓	x	x	✓	x	✓	x	x	✓	x	✓	x	x	~	✓
Ka’opua et al. (25)	✓	✓	✓	✓	x	✓	x	✓	x	x	✓	✓	✓	x	x	✓	x	x	x
Knoche et al. (36)	✓	✓	✓	x	x	x	x	x	x	x	x	x	✓	✓	✓	x	x	x	x
LoGiudice et al. (29)	~	✓	✓	x	x	~	✓	x	x	x	x	x	x	x	✓	x	x	x	x
McElmurry et al. (19)	~	✓	x	x	x	~	x	x	x	✓	x	✓	x	✓	~	✓	x	~	✓
McEwen et al. (31)	~	~	x	✓	x	✓	✓	x	x	✓	x	✓	x	x	x	x	x	✓	x
Nicolas et al. (30)	✓	✓	✓	x	x	✓	x	~	x	x	~	x	–	–	–	–	–	–	–
Oser et al. (23)	~	✓	✓	x	✓	x	x	x	x	x	x	x	x	x	x	x	✓	x	x
Sanderson et al. (26)	~	✓	x	x	x	x	x	x	x	x	x	✓	✓	✓	x	x	✓	x	x
Taylor et al. (18)	✓	✓	~	x	x	x	x	x	x	x	x	✓	✓	✓	x	x	✓	✓	x
Ward and Brown (28)	✓	~	x	x	x	✓	✓	✓	x	x	✓	x	✓	x	x	✓	x	x	✓
Wong et al. (21)	✓	✓	✓	✓	✓	✓	x	x	x	✓	x	✓	✓	x	~	✓	x	✓	x
Yeung et al. (33)	~	x	x	✓	x	✓	x	x	x	✓	x	✓	x	x	✓	✓	~	x	x

*✓, included; ~, partially included; x, not included; –, not applicable*.

## Cultural Competency Intervention Strategies

The interventions in the included studies utilized various approaches to increase cultural competence in promoting health for the population groups being targeted. Three distinct categories of cultural competency service and program intervention strategies were identified in the literature: community-oriented strategies, culture-oriented strategies, and language-oriented strategies. The specific approaches taken within these categories differed depending on the study focus and the unique cultural context and needs of the target group. The majority of studies incorporated strategies from two or more of these three categories.

### Community-Oriented Strategies

The greatest diversity of approaches implemented across studies was within community-oriented strategies. These strategies were further categorized into community partnerships, community participation, community spaces, and community networks and media. The most common community-oriented strategy, utilized in 18 (18/22, 82%) of the interventions, was the participation of community members in the development and implementation of service-level interventions. Members of the community health workforce were the most frequently represented in studies (16–20), followed by general community members (18, 21–23) and community or church leaders (24, 25). Other community members involved in these interventions were cancer survivors and family members of survivors (25, 26), heart attack and stroke survivors (23), community volunteers (24), seniors/elders (17) and representatives (27), and clinicians from the target population (28).

Example 1 **Community participation**

Ka’opua et al. (25) evaluated a culturally tailored breast cancer screening educational intervention for Native Hawaiian women delivered through local churches. The local minister, church congregant liaisons, and church volunteers were involved in the delivery of the sessions, and breast cancer survivors and family members of survivors from the local community delivered testimonials (25).

Another commonly reported community-oriented strategy was the creation of community partnerships through the research process. Two studies identified taking a community-based participatory research (CBPR) approach (16, 25); another identified using a community participation framework to guide the research (21); and a further two discussed the involvement of a community steering committee (29) or advisory board (30). Other studies discussed community involvement such as being initiated by community leaders and maintaining a strong community engagement focus (24), thereby establishing partnerships with key community groups and stakeholders (30) and collaborating with community health departments (23).

Example 2 **Community partnerships**

Nicolas et al. (30) reported on the process of culturally adapting an evidence-based cognitive behavioural therapy group treatment for Haitian American adolescents diagnosed with depression. The community participation approach included creating an advisory board with representatives from various stakeholder groups and establishing collaborative partnerships with community mental health centers and schools. The community partners participated in all stages of the project playing a key role in the design, implementation, and evaluation (30).

Another innovative community-focused strategy was the use of non-clinical, community spaces to increase the acceptability and accessibility of health interventions for the target groups. Community spaces included local churches (25, 31) or other religious facilities (24), a community center (32), and a community health center (33) and tribal clinic (34).

Example 3 **Community spaces**

Chavez-Korell et al. (32) reported on a culturally adapted depression treatment for older Latino adults delivered through a community center located in an area with a high older Spanish-speaking, low-acculturated, and first-generation immigrant population. The target population had a strong sense of trust and identification with the center, and already attended regularly for a variety of services and programs. Additionally, some elderly community members lived onsite at aged care housing operated by the center (32).

Finally, there were three studies which utilized community networks and local media in the promotion and recruitment aspects of programs. This included the use of local, language appropriate radio and television, and print media (21, 23, 24, 32), announcements at local religious facilities (24, 32), and community meetings and events (21, 32).

Example 4 **Community networks and media**

Oser et al. (23) evaluated a heart attack and stroke symptom public awareness campaign for two Native American reservation communities. The campaign was delivered through local print and radio media channels and theater advertisements. Additionally, campaign material was included in local press releases, print inserts, and direct mailers and was featured on road signs at one community (23).

### Culture-Oriented Strategies

Various culturally oriented adaptations and strategies were implemented across the evaluated interventions, with the majority of studies including several different cultural aspects. The cultural adaptation most commonly reported was the inclusion of some aspect of the target group’s cultural values, beliefs, and practices/traditions (17, 20–22, 25, 27, 28, 30–32, 34), including things such as recognizing the role of extended family (25, 27), the involvement of family (21, 32), and the use of culturally relevant metaphors (25, 30). Several studies also integrated aspects of the target community’s religion/spirituality and culture (17, 24, 25, 28, 32, 34) and included culturally relevant activities congruent with the unique lifestyle preference of the targeted culture (28, 29, 31, 32). Finally, three Australian studies reported the development of interactive and visual intervention resources (16, 20, 35) as a strategy for increasing the cultural appropriateness of health promotion for aboriginal people.

Example 5 **Cultural strategies**

Arora et al. (17) reported the inclusion of religious/cultural artifacts in the clinic screening protocols. Before and after every clinic, ceremonies were held under the guidance of an invited spiritual leader from the community. “Smudge” ceremonies were held to purify the body and invite health into the participant. Open circles were held for participants to discuss physical, mental, spiritual, and emotional health issues and goals. A tepee was set up outside the clinic for attendees to gather to socialize and participate in more cultural activities (17).

### Language-Oriented Strategies

There were three primary forms in which interventions were made linguistically appropriate for target groups: full language adaptation, partial language adaptation, and the creation or translation of written and audiovisual resources. There were seven studies in which participants could choose to have the entire intervention delivered in a language other than English: three programs were delivered in Spanish by bilingual health professionals (19, 31, 32); one program was offered to South East Asian participants in English, Gujarati, Punjabi, Hindi, or Dari (24); two studies delivered the intervention for Chinese and Korean New Zealanders (21), and Chinese American (33) participants in their chosen language; and one study hired nurses fluent in Cree (17). In four studies, partial language adaptations appropriate for the population were implemented. For example, two studies with African-American people discussed utilizing colloquial language and limiting medical jargon (22), and the incorporation of relevant and meaningful language, such as naming the program “Oh Happy Day” after the popular gospel song (28). A further two studies included minimal language adaptations, through the use of native words in spoken and written aspects of the programs (25, 34).

In some studies, the creation or translation of written materials, such as forms and documents, or educational materials for program participants, was only one aspect of a broader program (19, 21, 27, 32). However, three studies reported a primary intervention as an audiovisual or multimedia resource developed or translated in the language/s of the target population. These interventions were as follows: an e-mental health application that was translated from English to Yolngu Matha for an aboriginal Australians of the Yolngu language group (16); a breast cancer education video in Navajo language with English subtitles for Native American women (26); and a dementia education resource in Australia translated from English into three aboriginal languages (18).

Example 6 **Audiovisual resources**

Taylor et al. (18) reported on the evaluation of a culturally and linguistically targeted dementia awareness pilot resource in three aboriginal languages (Warlpiri, Kriol, and Djambarrpuyngu) as well as English. The video was pilot tested and evaluated with aged care workers and service users, and community members to assess the effectiveness of the resource and evaluate the difference that culturally safe inter-communication can make toward dementia education (18).

## Cultural Competency Intervention Outcomes

There were two main types of outcomes using various indicator measures across the studies: intermediate health-care outcomes, including patient and staff satisfaction, service utilization/access, and retention/adherence/treatment rates; and improvements in health-related knowledge and behaviors, and health outcomes. While the included studies reported many positive outcomes, there were measurement and study quality issues which limit the interpretability and generalizability of results. There was a lack of validated measurement tools used to assess outcomes and inconsistent reporting of outcomes. Multiple outcomes were also reported in many studies. Even studies which evaluated the same indicator type measured and reported this in different ways. Furthermore, it is not possible to link outcomes directly with types of strategies because of the multistrategic nature of most interventions, and absence of attribution of outcomes to particular strategies.

The most common outcome reported in 12 of the 22 included studies (55%) was patient-perceived acceptability of interventions. Positive appraisal of interventions was reported in all studies, which addressed this indicator. Five studies reported directly on patient satisfaction (17, 22, 24, 27, 28), while the remaining seven reported a range of other outcomes related to program acceptability (16, 18, 21, 25, 26, 32, 36). Only one study used a validated satisfaction measurement tool (28), and only two reported an increase in satisfaction following interventions (17, 27). An additional two studies included measures of patient/participant trust along with satisfaction (17, 22). A further six studies evaluated the acceptability and usefulness of health interventions from the perspectives of health professionals (16, 18–20, 26, 35, 36).

Example 7 **Patient-perceived acceptability**

Guadagnolo et al., utilizing a scale developed by the authors to measure satisfaction with care and medical mistrust, reported a significant improvement in scores for satisfaction with health care following a patient navigation service but found no change in scores for medical mistrust (27).

Four studies reported on health access/utilization outcomes with only two providing comparisons to pre-intervention service utilization rates (17, 29). Seven studies provided results on retention/treatment/adherence rates with high retention rates reported across three studies (25, 28, 32).

Example 8 **Service access/utilization and treatment rate outcomes**

Arora et al. found an increase in program attendance from 20% pre-intervention to 85% 2 years into a culturally appropriate program for a remote dwelling aboriginal community in Canada (17).

Chavez-Korell et al. utilizing program data reported a 96.7% retention rate in a culturally adapted depression intervention for elderly Latino adults (32).

Yeung et al. demonstrated an increase in treatment rates of Asian-American patients diagnosed with major depressive disorder from 6.5% pre-intervention to 45% during the intervention (33).

Four studies reported outcomes related to health knowledge and awareness with three of these noting improvements from pre-intervention levels (16, 18, 23). While eight studies discussed some behavior change outcomes resulting from interventions because of a lack of information on measurement tools and outcomes, only one study reported significant improvements on this measure (32).

Example 9 **Health knowledge and behavior outcomes**

Davies et al. reported some improvements in Hepatitis B-related knowledge for one group evaluated (16), while Oser et al. found significant improvements in knowledge of heart attack and stroke warning signs and symptoms across two American Indian reservations following a culturally relevant health education campaign (23).

Chavez-Korell et al. reported statistically significant improvements in the physical functioning of participants from baseline to 6 months, as measured by the Physical Component Summary score on the Short-Form 12-Item Health Survey which has established validity with the target population (32).

Finally, there is a significant focus of the cultural competency literature on improving specific health-related outcomes. The review found some evidence of improved health outcomes across five studies. The presence of improved health outcomes has been demonstrated in past cultural competency literature. Unlike other reviews that found strong evidence of improved health outcomes for cultural appropriate diabetes interventions across the literature (6, 11), with limited evidence reported for other health conditions (6), this review found evidence for improvements in depression severity resulting from culturally adapted mental health interventions (28, 32), and positive outcomes for cardiovascular disease (24, 37) with only one study reporting on improved diabetes risk indicators (19).

Example 10 **Improved health outcomes**

Chavez-Korell et al. (32) found a 50% or greater reduction in depressive symptoms for 56.15% (73/130) of participants at 6 months and for 63.22% (55/87) of participants at 12 months. This study also showed preliminary results of a statistically significant improvement in overall quality of life (QOL). The measurement tools used were shown to be reliable and valid for use with older adults, Latino’s and Spanish speakers (32).

Ward and Brown reported a decrease from moderate to mild depression and improvements in QOL measures of physical health and mental health in their first pilot, and a decrease from moderate depression to no depression in their second pilot, utilizing measures which have been validated with African-American people (28).

Jones et al. found a statistically significant improvement in cholesterol measures in a community-based CVD screening risk intervention (24), while Houston et al. found substantial and significant improvements in blood pressure for patients with baseline uncontrolled hypertension when compared to the control participants (37). Finally, McElmurry reported improvements in blood glucose control measured by a statistically significant drop in levels of hemoglobin A_1c_ (HbA_1c_) <0.001 (19).

## Study Quality

Similar to other cultural competency reviews (6), we found that the overall methodological quality of studies was moderate to poor. Only 3 of the 22 papers (14%) were rated to be of strong study quality (20, 24, 37). A further 8 (36%) papers were rated at moderate (17, 18, 22, 23, 25, 27, 30, 32) and 11 (50%) were rated weak (16, 19, 21, 26, 28, 29, 31, 33–36). This lack of strong quality studies limits conclusive statements about the effectiveness of cultural competence interventions. There is evidence of the ability to make positive impacts on improving patient/client health outcomes; however, a lack of methodological rigor was common. There was a lack of properly controlled studies where the study outcomes can be attributed to the intervention only. Additionally, there was an over-reliance on self-report measures and a lack of objective evidence of intervention effectiveness, as well as a lack of properly validated measurement tools for assessing outcomes. Overall, the strongest evidence came from United States-based and Canadian studies with Australian and New Zealand lagging behind in terms of study quality.

## Discussion

**Figure 4:**
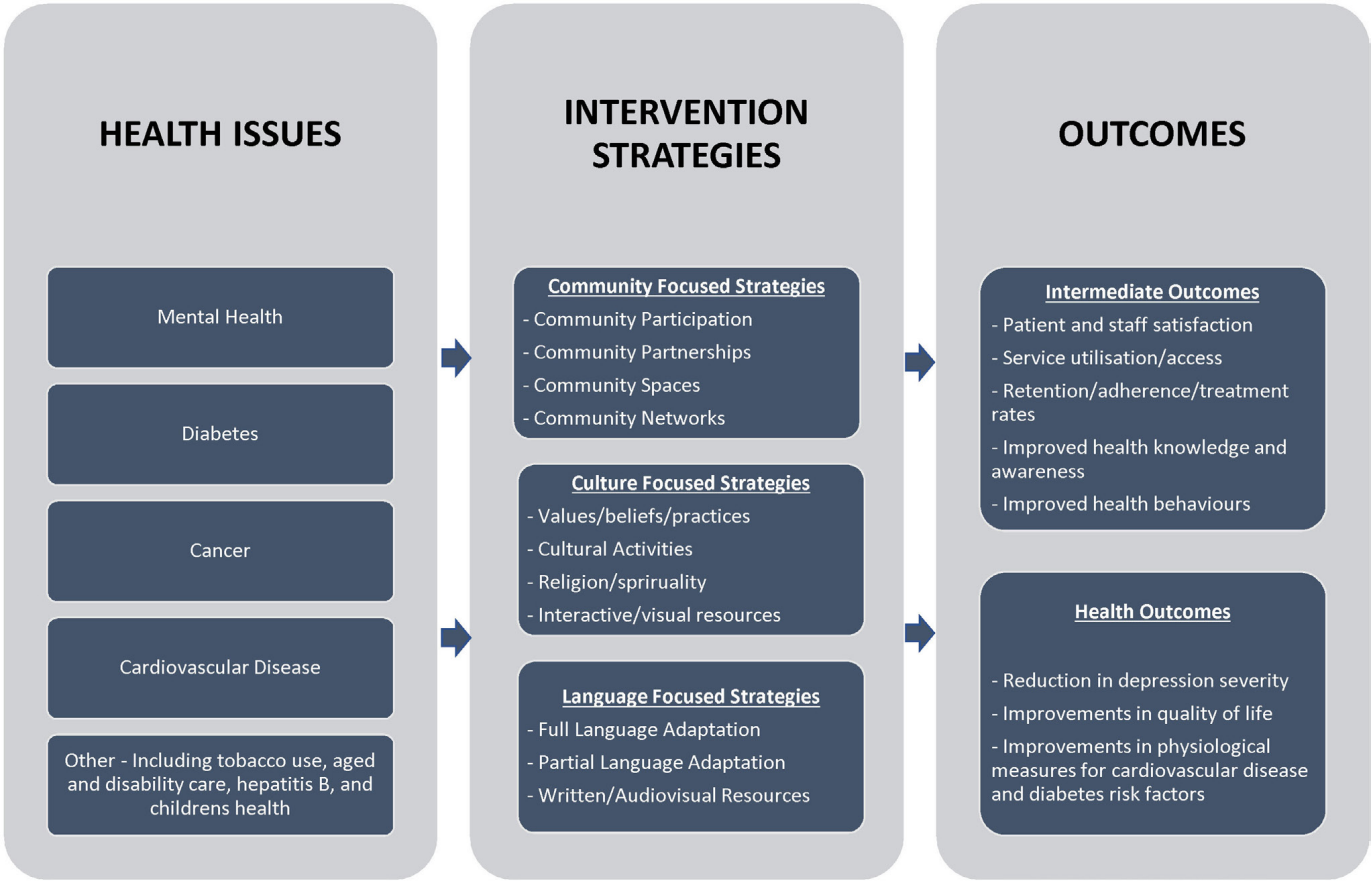
**Preliminary framework for health services and programs to improve cultural competency**.

Similar to what has been previously identified in the literature (6, 11), we found the interventions used across studies to be very heterogeneous in terms of target population, health issues and settings, intervention strategies, and outcomes. This variation reflects the complexity of cultural competency services and programs and their implementation in practice and research (6). Yet despite this heterogeneity, there are clear patterns across the included literature, in both intervention strategies and outcomes. These broad strategy and outcome types can help to inform future cultural competency services and programs. In Figure 4, we present a framework for planning, implementation, and evaluation of cultural competency services and programs based on the evidence from the reviewed intervention studies.

Consistent with health promotion evidence that multi-level approaches are required when designing appropriate interventions to address health-care disparities (38), studies used multiple intervention strategies including community-, culture-, and language-focused approaches. There were several community-focused strategies commonly implemented across the studies, the most common being the participation of community members in intervention implementation. This strategy is similar to what has been identified in the cultural competency literature over the past 15 years (9, 11). While community participation is important for cultural competency interventions, it needs to be distinguished from the kind of community-based partnerships recognized as key to addressing health disparities at the local level (2).

“The relative lack of involvement of diverse patients and communities in determining study issues, questions, designs, analysis and dissemination of results” is recognized as a major limitation in cultural competency research (p. xi) (11). The results from this review indicate some progression toward stronger community partnerships throughout the whole research process. However, there were only two studies which explicitly utilized a CBPR approach in project development, design, implementation, evaluation, and dissemination (39). Considering that working in collaborative partnership with community leaders and key stakeholders to build community capacity is identified as a health promotion core competency (40), this feature of program planning and implementation deserves greater attention in cultural competency initiatives within health promotion services and programs.

Another community-focused strategy of the included studies was the use of community resources, through both the use of community space and community networks. Health interventions conducted in non-clinical or community settings have received global research attention (41–46). In the reviewed evaluations, this approach was shown to be an effective strategy for reaching different population groups that typically do not access health services, especially mental health services. This intervention strategy demonstrates innovation on the part of health services, showing flexibility in approaches to increase the accessibility and appropriateness of services. Finally, the use of community networks in health service recruitment and promotion was a strategy not previously discussed in the cultural competency literature. This strategy was shown to be effective in engaging the target population and building community support for interventions.

The tailoring or adaptation of health interventions to be more congruent with the cultural beliefs, values, and practices of target groups is one of the most recognized and utilized strategies in cultural competency services and programs. However, the inclusion of cultural focused strategies has the potential to go beyond merely attempting to make health care more appropriate for communities into understanding the health benefits of cultural engagement. This is particularly pertinent for groups such as indigenous peoples who hold worldviews of health and well-being that link engagement in cultural activities with health benefits (47, 48). There is research evidence to link engagement in practices of caring for country to better health outcomes for aboriginal peoples in Australia (49) and engagement in traditional cultural and spiritual activities with increased alcohol cessation with Native American peoples (50).

The interconnection of spirituality and culture is also a potentially powerful resource for culturally appropriate health promotion, which was utilized throughout the included interventions. Spiritually based resources, which include values, beliefs, and practices based on a connection to a higher or sacred power have been correlated with the long-term survival of those with breast and other treatable cancers, and have been utilized as a resource for positive engagement of different populations with health services (51). The integration of spiritual and/or religious components is also consistent with the worldviews of many indigenous peoples and other cultural groups where health is understood holistically in all of its mind, body, emotional, and spiritual dimensions (52). The studies reviewed provide some innovative examples of cultural adaptation and engagement strategies utilized by cultural competency services and programs.

Clear and effective communication between health service providers and users is critical to quality and safe health-care provision. Therefore, access to health information and services in one’s primary language is essential for quality, accessible health care. Employment of native speaking health workers/educators/promoters is one important strategy (53), which was seen across the reviewed interventions. Interestingly, even when programs were made language accessible, it was not necessary that this was utilized by participants. For example, while Jones et al. offered the cardiovascular disease risk factor screening program in multiple languages based on the target population, 71% of participants chose to have the intervention delivered in English (24). Similarly, Taylor et al. found that while participants found it useful to have dementia education resources in local languages, and this helped to build participant engagement, they also appreciated having the resource in English and considered it to be more important to be able to have discussions in language after viewing the resource (18). These studies both highlighted a point that was stated throughout many studies. Language accessibility is about having the *choice* to have interventions delivered in participants’ preferred language.

When working with people with worldviews divergent from the biomedical model, language accessibility needs to go beyond the use of interpreters and translators (54). As noted by Vass et al. (p37), “while words and worldview concepts vary between indigenous nations, the principles of working in-depth in language and through the indigenous worldview are likely to have relevance to any indigenous groups who do not speak English as a first language and who do not have a biomedical or Western worldview” (55). For example, miscommunication has been extensively documented in interactions between health-care providers and aboriginal Australian people accessing health care, related to a lack of shared understanding around basic health concepts (53–56). When working in cross-cultural spaces, an extensive exploration of the meaning of words in health and specific health topics is needed, as is the development of health interventions and information which incorporates and builds on both traditional and contemporary indigenous health frameworks (55). Some of the included studies addressed such issues of intercultural communication in the context of worldview differences. Some studies also detailed the testing of translated program resources for appropriateness with the target population, while others did not. When reported, different levels of detail around the process and quality of the translation were provided in the included studies. This issue of differences in fundamental concepts of health and comprehension of health information is one area that deserves further attention in cultural competency program design and implementation.

The included studies utilized varying levels of integration of community-, cultural-, and linguistically focused cultural competency strategies. Okamoto et al. proposed a continuum model for understanding the level of adaptation involved in cultural competency prevention interventions, starting with non-adapted/surface-level adaptations, to deep-structure adaptations and, beyond this, culturally grounded interventions (57). This concept of a continuum of cultural adaptations in health programs has predominantly been discussed in relation to the adaptation of evidence-based treatments (EBT) (57, 58). Nonetheless, it has relevance beyond EBT to the design and implementation of other cultural competency interventions. The literature points out that surface and deep-structure adaptations can be very effective for many interventions with different groups. However, for some groups, particularly indigenous peoples, there is a greater need for culturally grounded approaches which are embedded in and created from the specific cultural viewpoint and needs of communities from the outset (57).

To improve the evaluation quality of cultural competency services and programs, greater attention on the use of appropriate, and where available, validated measurement tools is needed. The included studies provide useful evidence on intermediate outcomes such as satisfaction levels and service utilization rates. Nevertheless, the presence of key methodological flaws, such as a lack of pre-intervention comparisons, diminishes the strength of outcome data on intermediate health outcomes. In contrary, the studies demonstrating improved health outcomes generally used fairly rigorous study designs with appropriate measurement tools. This kind of attention to study quality is needed to measure intermediate and health outcomes, both of which are important indicators of intervention success.

Viewed together, these studies illustrate a wealth of potential approaches to inform future health promotion services and programs to improve culturally competency. The similarities in intervention strategies seen across these studies can be useful when planning cultural competence interventions in health services and programs. However, we would caution against the assumption that what works in one context is appropriate for others, even with the same cultural or ethnic group. The types of adaptations and strategies that are appropriate will differ according to the unique needs and circumstances of each community and target group. This reaffirms the importance of community partnerships to ensure that health interventions are responsive to the local context in which they are placed.

## Limitations

The publications in this review were identified with a non-exhaustive search strategy designed to produce peer- and non-peer-reviewed health studies that evaluated cultural competence interventions in health services. Therefore, it is possible that some relevant publications were not found. However, given the search strategy, including electronic databases, websites/clearinghouses, and reference lists of reviews, it is highly likely that the studies reviewed are representative of published cultural competence research from the United States, Canada, New Zealand, and Australia. Additionally, because of the breadth of this field, only studies that explicitly addressed improving cultural competency were included. This strategy possibly excluded studies that might have implicitly aimed to increase cultural competency. For the development of the literature base on the effectiveness of various interventions to improve cultural competency, it is important that studies explicitly address this in their aims and measures.

Another limitation occurred within the frameworks used to study quality. In the context of cultural competency, it is important to acknowledge that judgments of study quality are based on the scientific paradigm, which stresses the importance of “objectivity” (59) with subjective sources of knowledge considered to be less reliable (60). If cultural competency calls for the examination of worldview differences and a shift in power relations between researchers and communities/participants, this necessarily includes an examination of research methodologies and assumptions which marginalize other approaches and values of knowledge generation. Indigenous and other ethnic minority groups might value other quality criteria such as the extent of indigenous/ethnic group leadership and participation in the research and indigenous/ethnic group prioritization of the research focus (61).

To determine whether and to what extent culturally competent service provision enhances outcomes of services and treatment, it is essential that cultural competency is accurately assessed (8). However, a lack of systematic tools and approaches for measuring the presence, level, and contribution of cultural competency interventions to quality health care continues to weaken the growing evidence base (8, 62). Additionally, there was a preponderance of intermediate and short-term health outcome reported. Further research is needed into longitudinal, population-based studies to determine the overall impact of cultural competence interventions on population health and health disparities among groups.

## Conclusion

The included studies demonstrate a growing evidence base for the impact of health promotion services and programs to improve cultural competency on intermediate and health outcomes. Nonetheless, because of methodological issues related to appropriate indicators and study design, it cannot be definitively concluded what types of interventions produce what types of outcomes with particular populations. Interventions need to be based on the evidence available for what works with different populations and health issues as well as the desires of the community/target population. The primary lesson from reviewing the strategies and approaches to culturally tailoring or developing culturally grounded health interventions for minority population groups is that each needs to be consistent with the unique cultural needs and characteristics of target populations and need to be embedded in context and community.

## Author Contributions

CJ is the primary author and was responsible for the data extraction of the search update and the writing of the final review manuscript. RB completed the data extraction for the first search and authored a paper on the larger review in 2014 which informed this review. The authors JM, RB, and CJ have all contributed in the following ways. All authors have: contributed significantly towards the development of the review concept and structure; been involved in drafting the paper and critically reviewing content during the editing process; previewed the final version of the review; and approved it for publication. The authors are assured of the accuracy and integrity of the review and agree to be accountable for all aspects of the publication manuscript.

## Conflict of Interest Statement

The authors declare that the research was conducted in the absence of any commercial or financial relationships that could be construed as a potential conflict of interest.
