# Supplementary material for: The Implementation and Evaluation of Health Promotion Services and Programs to Improve Cultural Competency: A Systematic Scoping Review
Source: Front Public Health. 2017 Feb 27;5:24. doi: 10.3389/fpubh.2017.00024 (PMC5327788; doi:10.3389/fpubh.2017.00024)
Supplement: Supplementary file 1 [file Table_1.DOCX]

| **Service-level cultural competency health promotion interventions** | | | | | | | |
| --- | --- | --- | --- | --- | --- | --- | --- |
| **Author, year and type of publication** | **Country where developed and target population** | **Health issue, intervention setting, and participants** | **Intervention type** | **Study design** | **Outcome measure or indicator** | **Health care outcomes assessed** | **Study quality** |
| **Aroa et al., 2013**  Journal Paper | Canada  Aboriginal Canadians | Diabetes  Community-based tribal clinic  5 patients, 2 program administrators, 1 hospital nurse, 1 nurse from Remote community, and 1 spiritual liaison | Culturally-sensitive diabetic retinopathy tele-ophthalmology screening program | **Quantitative:** clinic attendance rates;  **Qualitative**: interviews with attendees and key stakeholders | Clinic attendance rates and self-reported patient satisfaction | Increased appointment attendance from 25% to 85%. Qualitative accounts of increased patient satisfaction, trust towards the health-care team, and increased communication amongst participants. | Moderate |
| **Browne et al., 2014**  Journal Paper | Australia  Aboriginal Australians | Diabetes  State-wide: Aboriginal Community Controlled Health Service  66 Aboriginal health workers completed survey | An Aboriginal-specific diabetes prevention and management resource | Online evaluation survey to assess the appropriateness of the tool | Level of usage with Aboriginal community and confidence in discussing diabetes | 89% of health workers felt more confident in their ability to discuss diabetes with their community; 70% of workers had used Feltman with the community. | Weak |
| **Chavez-Korell et al. (2012)**  Journal Paper | United States  Latino older adults | Depression  Community-centre  130 participants from baseline to 6-months  87 participants from baseline to 12-months | Culturally adapted depression treatment program | Multiple time-series design | Depressive symptoms, physical functioning, and quality of life. | Overall, 56.15% (73 of 130) of participants have shown a 50% or greater reduction in depressive symptoms from baseline to 6 month, and 63.22% (55 of 87) of participants have shown a 50% or greater reduction in depression symptoms from baseline to 12 months.  Preliminary data also indicated statistically significant improvements in the physical functioning (from 36.26 to 38.49, *p* _ .01) and overall QOL (from 6.12 to 7.36, *p* _ .001) for UNA participants from baseline to 6 months. | Moderate |
| **Davies, 2015**  Journal Paper | Australia  Aboriginal Australians | Hepatitis B  Community settings  Indigenous people and communities in Northern Australia | Culturally appropriate bilingual electronic app about Hepatitis B | Participatory action research  Pre-post questionnaire | Improvement in Hep-B related knowledge, and views of acceptability and ease of use | Initial evaluation demonstrated significant improvement in Hep-B related knowledge, and strongly positive views of acceptability and ease of use | Weak |
| **Dingwall et al.,**  **2015** | Australia  Aboriginal Australians | Mental health  15 Mental health service providers in the NT | Culturally-tailored e-mental health resource for service providers working with Aboriginal and Torres Strait Islander people | Semi-structured interviews | Perceived barriers and enablers, acceptability, feasibility, engagement, appropriateness, perceived impact and degree of support required to use the app | Perceived as acceptable, feasible, and appropriate. Perceived as culturally relevant, and to have had a positive impact on the engagement process, helping to break down barriers and open up conversations. Constraints included; IT accessibility, time and language. | Strong |
| **D’Silva et al., 2011**  Journal Paper | United States  Native Americans | Tobacco use  Reservation and pharmacy setting  317 community participants | Tailored smoking cessation group and individual programs | Pre-post | Program satisfaction; Tobacco use; and quit rates | 90 day follow up. 47% reported abstinence. Missing = smoking analysis yielded a 21.8% quit rate. Continuing smokers cut their quit rate by half, and 88% reported an increase in self-efficacy for their next quit. | Weak |
| **Guadagnolo et al. 2011** | United States  Native Americans | Cancer treatment  Hospital  52 Native American cancer patients | Culturally specific program: patient navigation by trained culturally competent staff (training in Native American patients’ beliefs and cultural practices | Pre-post survey, no control group | Medical mistrust, patient satisfaction | Statistically significant improvements in levels of patient satisfaction (P<0.0001) but not medical mistrust | Moderate |
| **Houston et al. (2011)** | United States  African Americans | Cardiovascular disease  hospital setting  230 African American patients with hypertension | A culturally appropriate interactive storytelling intervention | RCT across three time series | Differential change in blood pressure | The intervention produced substantial and significant improvements in blood pressure for patients with baseline uncontrolled hypertension. | Strong |
| **Jandorf et al.,**  **2013**  Journal Paper | United States  African American patients | Colonoscopy screening  Primary care clinic  240. Pro-PN (n=106) or Peer-PN (n=134). | Professional patient navigator v´s peer patient navigator | RCT | Colorectal Screening adherence rates, patient satisfaction and navigator trust | No significant differences in SC adherence rates between Pro-PN (80.0%) and Peer-PN (71.3%) (P=0.178). Participants in both groups reported high levels of satisfaction and trust. | Moderate |
| **Jones et al.,**  **2013**  Journal Paper | Canada  South Asian Canadians | Cardiovascular disease  Local religious facilities  238 participants in initial screen, 99 presented for re-screening | Culturally-adapted, community-based Cardiovascular Disease (CVD) risk factor screening program | BP tests; measure of total cholesterol/high density lipoprotein (TC/HDL); and questionnaires | Health-system follow up, program satisfaction, and changes in risk factor measures from baseline | 82% of participants had accessed health care providers; 80% very satisfied and 20% satisfied with program; modest and significant improvements in cholesterol measures. | Strong |
| **Ka’opua et al., 2011**  Journal Paper | United States (Hawaii)  Native Hawaiians | breast cancer screening  Church-based  198 participants across 12 sites | Mammography – culturally tailored screening | RCT  Moderate | Protocols, procedures and processes; Knowledge, attitudes and practice | Recruitment exceeded targets and retention rates good, satisfaction high, positive outcomes are suggested | Moderate |
| **Knoche et al.,**  **2012**  Evaluation Report | Australia  Aboriginal Australians | Children’s health  Hospital  144 patient/families who accessed case management | A multi-strategic model of care to improve patient access and provide culturally appropriate care | Focus groups, individual interviews (families of patients, Wadja staff, and staff from Aboriginal CC and mainstream health organisations), clinic audits, and key document analysis | Accessibility, cultural appropriateness, patient satisfaction, referrals, employment | Positive feedback from families regarding care experiences; improved accessibility and cultural appropriateness; Increased number of patients seen over evaluation period; high-levels of patient satisfaction; increase in referrals to external agencies, especially Aborignal CC health services; increased cultural awareness activities; and increase in Aboriginal staff employed at hospital | Weak |
| **LoGiudice et al.,**  **2012**  Journal Paper | Australia  Aboriginal Australians | Aged and disability care  Community setting  11 clients and carers, 6 service providers, 6 project staff, and 2 community council members | Locally designed community service model of care | Evaluation at baseline then 6 and 12 months | Access and services provided | Increase in people receiving care (from 8 to 22), and an increase in services provided from 140 at baseline month to 2356 at 12 month evaluation month. | Weak |
| **McElmurry et al. (2009)**  Journal Paper | United States  Limited English Proficient (LEP) Latino patients | Diabetes  5 Ambulatory care sites across a health service network  392 patients | Culturally tailored diabetes education delivered by Community Health Workers | Multiple time-series | haemoglobin A_1c_ (HbA_1c_) levels, appointment attendance, self-reported blood glucose control behaviours | Improvements in blood glucose control as measured by a drop in HbA_1c_, and increase in self-reported blood glucose self-monitoring.  Increased appointment attendance in relation to increased HP contact. | Weak |
| **McEwen et al., 2010**  Journal Paper | United States  Mexican Americans | Diabetes  Local church and participants homes  23 participants | Culturally tailored diabetes self-management social support intervention, including bilingual, bicultural certified diabetes educator (CDE) | Pre-post, self-report survey | Behaviour (nutrition, physical activity, diabetes distress), knowledge, physiological measures (HbA1c and BMI). | Increase in diabetes self-care activities | Weak |
| **Nicolas et al. (2009)** | United States  Haitian American adolescents | n/a | **Intervention**  Culturally adapted cognitive behavioural therapy intervention to address depression | Qualitative analysis through focus groups | Participants perspectives on the cultural appropriateness of the intervention | n/a | Moderate |
| **Oser et al.,**  **2013**  Journal Paper | United States  Native Americans | Heart attack and stroke  Community setting  659 interviews from Rocky Boy’s reservation (n = 292, baseline survey; n = 367, post campaign survey). 1,154 interviews at Fort Belknap (n = 354, baseline; n = 400, post campaign; n = 400, post maintenance). | **Intervention** Culturally specific public awareness campaign for signs and symptoms of heart attack and stroke | Before and after telephone surveys | Recognition and knowledge of heart attack and stoke warning signs | Knowledge of 3 or more heart attack warning signs and symptoms increased on 1 reservation from 35% to 47%. On the second reservation, recognition of 2 or more stroke signs and symptoms increased from 62% to 75%, and the level of awareness remained at 73% approximately 4 months after intervention. Intent to call 9-1-1 did not increase in the heart attack campaign but did improve in the stroke campaign for specific symptoms. Recall of media campaigns on both reservations increased significantly from baseline to post campaign. | Moderate |
| **Sanderson et al, 2010** | United States  Native Americans | Breast Cancer  14 Native American women with breast cancer; 26 health professionals | Culturally specific program: 12 min culturally specific breast cancer educational video for health professionals treating women. | Post-test survey only, no control group | Knowledge, attitudes and beliefs | Knowledge, attitudes and beliefs post-video and 6 months (follow up 100%) | Weak |
| **Taylor et al**  **2012**  Journal Paper | Australia  Aboriginal Australians | Dementia  Community setting  Indigenous aged care workers, community members and aged care service users (n=26), and health care professionals and service coordinators (n=5) | Targeted dementia awareness resource in Aboriginal languages | Qualitative: focus groups and semi-structured interviews | N/A | Increased dementia awareness among target audience and broader community; Changes in attitude and behaviour towards people with dementia. | Moderate |
| **Ward et al. (2014)** | United States  African American | Depression  50 African American adults | Culturally adapted depression intervention | Multiple time-series design | Recruitment and retention rates  Depressive symptoms | Pilot 1: 73% of participants completed the intervention  Pilot 2: 66% of participants completed the intervention  In both groups, participants showed a statistically significant decline in depressive symptoms and were very satisfied with the intervention. | Weak |
| **Wong et al., 2010**  Journal paper | N.Z.  Asian New Zealanders | Tobacco use  Home, workplace and clinic based delivery  104 clients | Asian specific service model for Asian smokers | Post-test only  Weak | Client satisfaction, appointment attendance, and quit rates | Reported satisfaction with program; 97% appointment attendance; Self-reported quit rate was 72% at 1 month, 53.8% at 3 months, and 40.9% at 6 months. There was an 18% increase in smoke free homes following the intervention. | Weak |
| **Yeung et al. (2010)** | United States  Chinese Americans | Depression  Community health centre  100 participants | Culturally sensitive collaborative treatment for depression | RCT  Program data on treatment rates | Treatment engagement and treatment response | Patients in the care management and usual care groups did not differ in terms of their outcomes. CSCT resulted in a nearly 7-fold increase in treatment rate among depressed patients in primary care. | Weak |
