# Supplementary material for: The Implementation and Evaluation of Health Promotion Services and Programs to Improve Cultural Competency: A Systematic Scoping Review
Source: Front Public Health. 2017 Feb 27;5:24. doi: 10.3389/fpubh.2017.00024 (PMC5327788; doi:10.3389/fpubh.2017.00024)
Supplement: Supplementary file 2 [file Table_2.DOCX]

| ***Publication*** | ***Intervention*** |  | ***Cultural Competence Intervention Strategies*** | | | | |
| --- | --- | --- | --- | --- | --- | --- | --- |
|  |  |  | ***Community*** |  | ***Cultural*** |  | ***Language*** |
| **Arora et al. (2013)** | A Diabetic retinopathy tele-ophthalmology screening program for Aboriginal Canadians, delivered in a culturally-sensitive community-based clinic. |  | The clinic was set up at the community-level in a remote clinic serving a predominantly Cree community. Community members were involved in the delivery of the program, including staff and Elders/leaders. |  | Religious/cultural artefacts were included in the clinic screening protocols. Before and after every clinic, ceremonies were held under the guidance of an invited spiritual leader from the community. ‘Smudge’ ceremonies were held to purify the body and invite health into the participant. Open circles were held for participants to discuss physical, mental, spiritual and emotional health issues and goals. A tepee was set up outside the clinic for attendees to gather to socialise and participate in more cultural activities. |  | Nurses fluent in Cree were hired from local communities. |
| **Browne et al. (2014)** | An Aboriginal specific diabetes prevention and management resource for Aboriginal Health Workers. |  |  |  | The education resource was made culturally appropriate by being visual and interactive. |  |  |
| **Chavez-Korell et al. (2012)** | A culturally adapted depression treatment for Latino older adults and elders (aged 60 years and older) called *Un Nuevo Amanecer (*A New Dawn). |  | The treatment was delivered through a non-mental health community centre setting which the local Spanish-speaking, low-acculturated, first-generation immigrant older adult Latino target population has a strong sense of trust and identification with, and already attend regularly for other activities.  Information about the intervention was disseminated in the Latino community through announcements at area churches; on local Spanish radio shows; coverage on local Spanish public television; and informational sessions at community festivals, health fairs,  and social gatherings. Information was also disseminated by hosting Latino-style bingo  games at older adult public housing buildings  and at older adult day programs serving Latino elders. |  | Cultural sensitivity and cultural humility were used in all recruitment efforts, screenings, treatment planning, interventions and interactions with participants and their families. Latino cultural values of *familismo* (family orientation and connectedness), *personalismo* (recognition of the individual within a larger social and familial context), *respeto*, *dignidad*, *espiritualidad* (strong sense of spirituality and/or religiosity), and the gender roles of *machismo* (male gender role) *and marianismo* (female gender role) were carefully considered in treatment conceptualization, planning, and intervention.  Culturally sensitive and appropriate activities were used in the Behavioural Activation and Problem Solving Treatment exercises to improve participants’ buy-in to using these tools, to increase the relevance of the exercises, and thus to improve treatment outcomes. Many of the Behavioural Activation pleasant activities  involved family and community activity. When using PST, the DCMs were careful not to impose their own value systems in the problem-solving exercises and honoured the client’s solutions. |  | All staff involved in the intervention and research were culturally competent, bilingual and bicultural. All forms and documents were translated to Spanish and all treatment and communications were delivered in participants preferred language. |
| **Davies et al. (2015)** | A culturally appropriate bilingual application about hepatitis B was developed for remote dwelling Indigenous Australians in Arnhem Land. |  | A Participatory Action Research (PAR) framework was used in the development of the app.  The original motivation for the project came from staff at the local community health clinic. This lead to a collaborative research project involving the regional Aboriginal Community Controlled Health Service, the Royal Darwin Hospital Liver Clinic, the Menzies School of Health and a local graphic design and software company. |  | Culturally appropriate and respectful images were used, using real people. There was an emphasis on interactive pictures and less text.  There was consideration for culturally appropriate gender representation and a separate ‘Women’s Business’ section to discuss issues around mother to child transmission of Hep B. |  | The app can be used in either English or Yolngu Matha (the most common language in East Arnhem Land). The app was both forward translated from English to Yolngu Matha, then back translated to check the accuracy of the translation. The emphasis was on conceptual and cultural equivalence rather than linguistic equivalence. |
| **Dingwall et al. (2015)** | A culturally-tailored e-mental health resource for working with Aboriginal and Torres Strait Islander people in Australia |  | The resources were originally developed with local Aboriginal mental health workers. |  | The program was developed through exploring local Indigenous perspectives of mental health promotion and with recognition of the holistic nature of wellbeing.  The app uses a highly visual, interactive format which is more culturally appropriate for Indigenous people. |  |  |
| **D’Silva et al. (2011)** | A culturally specific curriculum for tobacco dependence treatment among Native Americans. |  | Adaptations to the American Lung Association’s “Freedom from Smoking” program were made based on suggestions from key community stakeholders.  The program included community outreach and education. Sessions were held at the tribal clinic located on the reservation. |  | The curriculum was modified to incorporate the cultural and historical characteristics, values, and traditions of the community.  Adaptations included the additional of Ojibwe stories and teachings on how to use tobacco as a sacred item in ceremonies and offerings of prayer, distinguishing between sacred tobacco use and commercial tobacco abuse |  | Adaptations included the additional of Ojibwe language. |
| **Guadagnolo et al. (2011)** | A culturally tailored Patient Navigation (PN) program for American Indians (AI) undergoing cancer treatment. |  | The navigation program included community research representatives who work closely with the hospital staff and are imbedded in the surrounding AI communities, and who serve as liaisons between the cancer centre, hospital-based PN and patients or tribal governments. |  | All AI cancer patients were offered PN services where they would be assisted in navigating the medical system by trained, culturally-competent staff, who have specific training regarding American Indian beliefs and cultural practices. This included the role of the extended family network and involvement in care. |  | Patient education materials were translated into the Lakota language by staff members who were either closely connected with or are members of the American Indian communities served by the program. |
| **Houston et al. (2011)** | A culturally appropriate interactive storytelling intervention in the form of DVD’s to improve blood pressure among African American patients with hypertension. |  | Patients from within the local community who represented a range of experiences were selected to share their stories about a range of issues related to treatment of hypertension. These stories were edited and made into a DVD for the intervention group. |  |  |  |  |
| **Jandorf et al. (2013)** | A culturally targeted patient navigation (PN) program for African American screening colonoscopy (SC) patients. |  | Community-based peer navigators (Peer-PN´s) were recruited for the delivery of the PN program. |  | The socio-cultural approach addressed health-related issues in the context of the broader social and cultural values and concerns of African Americans. |  | The linguistic approach focused on using colloquial language and limiting medical jargon. |
| **Jones et al. (2013)** | A feasibility study of a community-based, culturally adapted cardiovascular disease risk factor screening program for South Asian (SA) Canadians. |  | The researchers were initially approached by SA community leaders requesting the implementation of the program. These same community leaders chose six local religious facilities as screening locations. Participation was invited by community leaders through announcements at the facilities, and notices in local community newsletters, radio and television.  The screening program was delivered by lay trained volunteers identified by community leaders. |  | The intervention was conducted at local culturally relevant religious facilities attended by the SA community. |  | Program participants were initially screened for their language of choice then directed to the appropriate volunteer for further assessment. All program activities were delivered in the participants language of choice (English, Punjabi, Hindi or the Dari dialect). |
| **Ka’opua et al. (2011)** | A feasibility study of a culturally tailored breast cancer screening educational intervention for native Hawaiian women in rural churches. |  | The intervention was designed using a Community-based Participatory Research (CBPR) approach.  The intervention occurred at local churches were a special worship service was held focused on providing breast health education. The local minister, church congregant liaisons and church volunteers were involved in the delivery of the sessions, welcoming people, providing program print material, and introducing the program and reading relevant scriptures. Breast cancer survivors and family members of survivors from the local community delivered testimonials. |  | The intervention was a church-based education program integrating both Native Hawaiian and Judeo-Christian values and practices. It emphasised Hawaiian cultural strengths, including those related to spirituality and the extended family system. Native Hawaiian language and metaphors were used to deliver the program messages. |  | Native language and words were used throughout the sermon, and in the spoken and written aspects of the intervention. |
| **Knoche et al. (2012)** | A New Model of Care, *Wadja*, to improve cultural safety of services and cultural competency of the Royal Children’s Hospital in Melbourne Australia. The model is a multi-level intervention including direct health services, staff training and professional development. |  | Local Aboriginal people were employed as staff in the Wadja clinic. A participatory approach to the evaluation was used involving Wadja staff. Partnerships were created with local Aboriginal organisations. |  |  |  |  |
| **LoGuidice et al. (2012)** | A locally designed and culturally appropriate pilot community service model of care for aged and disabled people in a remote Aboriginal community in Australia. |  | The model was implemented based on extensive community consultation with older people, families, carers, community members and stakeholders. The project was co-managed by a steering committee consisting of representatives from the community council, government and non-government services. The coordinator and workers were employed from within the local community. |  | Culturally relevant respite activities included fishing, visiting country, and art. |  |  |
| **McElmurry et al. (2009)** | A culturally appropriate diabetes education by Community Health Workers (CHW’s) for Limited English Proficient (LEP) Latinos. |  | CHW’s delivering the education are trusted and respected community members. |  | CHW’s have inside cultural knowledge regarding common health beliefs and practices in their community and therefore have the capacity to deliver culturally appropriate health education. |  | CHW’s adapted and translated printed diabetes education resources for use with patients, and conducted education and consultations in Spanish in group and individual sessions. |
| **McEwen et al. (2010)** | A culturally tailored diabetes self-management social support intervention for Mexican American adults with type 2 diabetes. The intervention was developed by a bilingual, bicultural certified diabetes educator. |  | Intervention participants met at neighbourhood church that was accessible by public transport for the group sessions. Individual sessions were delivered in participants homes. |  | Cultural tailoring included the use of Spanish language, the inclusion of dietary and physical activity preferences common in the border region of México, and social support congruent with the traditional Méxican cultural values of *simpatía* (kindness, politeness), *respeto* (respect) , and *personalismo* (formal friendliness). |  | The intervention was delivered in Spanish by bilingual ‘promotoras’ trained in the intervention. |
| **Nicolas et al. (2009)** | A research process of culturally adapting an EBT group cognitive behavioural therapy (CBT) intervention for use with Haitian American adolescents diagnosed with depression. |  | Creation of an Advisory Board with representatives from various stakeholder groups. Established community partnerships with community mental health centres and schools to discuss and get feedback on constructs of depression and their relevance to Haitians. As a result a new framework was developed that incorporated the specific cultural ideas of the Haitian community. Community partners participated in all stages of the project from the design to the implementation and evaluation of the project. |  | The intervention manual was adapted based on feedback from focus group participants to reflect the cultural beliefs and values of Haitian adolescents. Feedback was provided on the cultural relevance of metaphors and pictures used in the manual. |  | Participants were asked to evaluate the language and metaphors used in the intervention manual for accuracy in reflecting the culture of Haitian youth. |
| **Oser et al. (2013)** | A culturally specific public awareness campaign for the signs and symptoms of heart attack and stroke targeting two rural American Indian (AI) communities in Montana. |  | The campaign was developed and implemented in collaboration with two tribal health departments.  Focus groups were held on each reservation to obtain feedback from AI community members about sample themes of culturally appropriate advertisements. |  | A key role of the tribal health departments was to help convey each tribe’s unique cultural norms.  On the basis on input from focus group participants, print materials were developed to feature local residents and/or topography from each reservation. |  |  |
| **Sanderson et al. (2010)** | A culturally specific video designed to educate Navajo women about breast cancer treatment options. |  | The video was created by a Navajo breast cancer survivor. |  |  |  | The video was in Navajo language with English subtitles. |
| **Taylor et al. (2012)** | Culturally and linguistically targeted dementia piolet resource for Aboriginal Australians. |  | Pilot study tested the meaning and appropriateness of the resource with community members and aged care workers in the targeted communities. |  |  |  | The dementia awareness resource was translated into three Aboriginal languages. |
| **Ward & Brown (2015)** | A culturally adapted depression intervention for African American adults experiencing depression. |  | African American clinicians delivered the treatment intervention. |  | The intervention was based on the Afrocentric paradigm Nguzo Saba, which is comprised of seven humanistic principals: unity, self-determination, collective work, and responsibility, cooperative economics, purpose, creativity and faith.  The intervention incorporated African American cultural beliefs, and common beliefs regarding risk factors for depression among African American people, as indicated in previous research. Preferred coping behaviours were included such as use of religious coping and preference for group counselling. |  | Language relevant to African American people was used in adapting the treatment intervention. It was named ‘Oh Happy Day’ after the popular gospel song, with strong anecdotal support for this name. |
| **Wong et al. (2010)** | Asian Smokefree Communities (ASC) pilot program. A community-focused, Asian-specific smoking cessation and smoke free environments intervention for New Zealand Asian migrants. |  | The intervention was developed by a group of Asian and non-Asian tobacco control, smoking cessation, research and health promotion experts who worked closely with local Asian communities. A community participation framework was formulated to demonstrate the core principles of the program. The program was promoted extensively through Asian community networks and media such as the Asian language radio, television and print news media and Asian community meetings. A Chinese coordinator and a Korean coordinator were recruited from the local community. |  | A family and culture specific approach was used with the intervention targeting and being accessible to whole families if desired. |  | The Chinese and Korean coordinators delivered the intervention in participants preferred language. An interpreter service was available for program participants who spoke other languages.  Culturally appropriate resources were either created in the target languages, or translated and tested for appropriateness before use. |
| **Yeung et al. (2010)** | A culturally sensitive collaborative treatment to improve the recognition, engagement and treatment of depressed Chinese Americans in primary care. |  | Participants were recruited from a Community Health Centre which serves predominantly Chinese American patients with financial, language, and cultural barriers to health care. |  | Patients illness beliefs were explored through a set a questions derived from Kleinman’s questions which provided information on how patients conceptualized their illness in their own language and words. With this information, the treating psychiatrist introduced information on depression in ways that were compatible with patients’ beliefs. |  | Written and verbal assessment were bilingual. Communication with patients throughout the intervention process was done by bilingual practitioners. |
